# Health and wellbeing status of the long-lived individuals of the Spanish LONGECYL cross-sectional study

**DOI:** 10.1186/s13690-024-01305-5

**Published:** 2024-05-20

**Authors:** Tomás Vega-Alonso, José Lozano-Alonso, Lorena Estévez-Iglesias, Ana Ordax-Díez, Enrique Arrieta-Antón, Ángel Díaz-Rodríguez, José-Luis Yañez-Ortega, Alejandro Santos-Lozano, Rocío Nuñez-Torres, María Perez-Caro, Gillermo Pita, Rosa Pinto-Labajo, María-Jesús Alonso Ramos, Rufino Álamo-Sanz, Andrés-C García-Montero, Anna Gonzalez-Neira

**Affiliations:** 1 Dirección General de Salud Pública. Consejería de Sanidad, Valladolid, Spain; 2 Gerencia Regional de Salud. Consejería de Sanidad. Area de Salud de Segovia, Segovia, Spain; 3 Gerencia Regional de Salud. Consejería de Sanidad. Area de Salud de Ponferrada, Ponferrada, Spain; 4 Servicio Territorial de Sanidad de Burgos. Consejería de Sanidad, Burgos, Spain; 5 European University Miguel de Cervantes, Valladolid, Spain; 6 Banco Nacional de ADN, Universidad de Salamanca, Instituto de Investigación Biomédica de Salamanca (IBSAL), Salamanca, Spain; 7 Unidad de Genotipado Humano-CEGEN, Centro Nacional de Investigaciones Oncológicas, Madrid, Spain; 8 Consejería de Sanidad. Dirección General de Salud Pública, Paseo de Zorrilla, 1, Valladolid, 47071 España

**Keywords:** Longevity, Health status, Quality of life, Activities of Daily Living, Spain

## Abstract

**Background:**

The increase in life expectancy and long-lived individuals is a challenge for public health and provides an opportunity to understand the determinants of longevity. However, few studies have addressed the factors associated with the health status and quality of life in a long-lived individual population. We described the perceived health, clinical status, quality of life, and dependency for activities of daily living in a representative population in Castile and Leon, Spain.

**Methods:**

A sample of 759 long-lived individuals aged 95 years and older was studied by the Health Sentinel Network of Castile and Leon (Spain) through a health examination and a structured questionnaire covering quality of life (EQ-5D-3), lifestyle habits, diet, working life and family health. A blood sample was taken for the study of biological and genetic markers. Chi Square and logistic regression OR with 95% confidence intervals were used to analyze the determinants of the long-lived individuals’ health status. The significant level for the bivariate analysis was established at 0.05.

**Results:**

Perceived health was good, very good or excellent in 64.2%, while only 46.0% had a quality-of-life index above 0.5 (ranging from 0 to 1) and 44.1% maintained acceptable independence for activities of daily living. Quality-of-life index was higher in the oldest, (OR 7.98 [2,32-27.41]) above 100 years compared to those under 98, and men had better values for independence than women (OR 2.43 [1.40–4.29]). Cardiovascular diseases were the most prevalent (85.5%), but neurological and mental diseases and vision problems had the highest impact on quality of life and independence.

**Conclusion:**

The long-lived individuals of Castile and Leon have a relatively well-preserved health status, although the perception of health is higher than that describing their quality of life and dependence. The quality of life was higher in the oldest age group and showed differences according to sex, with a better quality of life in men. Public health policies and programs should take in account the differences by sex and age as well as the prevention and control of the main conditions related with poor quality of life or dependence. Future research must include the interaction among genetic, socioeconomic, environmental, and other clinical factors in the quality of life and disability of long-lived individuals.

**Table:** 

**Text box 1. Contributions to the literature**
• Epidemiological data on older people’s quality of life are limited, particularly in representative population samples.
• Over 95 years old, the perceived health status is higher than the quality of life and the observed dependence. Men have a better quality of life than women.
• Neurological, mental diseases and vision problems reduce the quality of life and increase the dependence.

## Introduction

The increase in life expectancy of the population is accompanied by an increase in the number of long-lived individuals (LLIs) and is an enormous challenge for the healthcare system and for public health in general. In Spain, hospital admissions of Spanish centenarians increased by 121.5%, from 2004 to 2020 [1]. An study in the Unites Stated estimates that for survivors to age 85, more than one-third of the total lifetime cost on health will accrue in the remaining years of life [2]. The Spanish National Institute of Statistics predicts that there will be approximately 100,000 centenarians in Spain by the year 2050 [3]. However, it remains the uncertainty whether this increase in longevity comes at the cost of an increase in years of life in poor health or causes inequities and consequently, increasing lifespan should focus on healthy life expectancy through a shift from intervention towards preventive health avoiding increases in health inequality [4].

The study of very long-lived individuals aims to improve the knowledge of the genetic [5], environmental [6] and lifestyle factors associated with increased survival and quality of life [7, 8], which should guide actions addressing the health and social problems affecting this population. It has been said that the description of the health of the long-lived and the investigation of its determinants should be on the agenda of priorities for any public health policy in countries with aged populations [9]. Moreover, the United Nations Sustainable Development includes the goals to ensure healthy lives and promote well-being for all at all ages and to reduce inequality by gender and age [10].

Castile and Leon is a large Spanish region of 94,206 km^2^; in 2021, it had 2.38 million inhabitants, 26.12% of whom were over 65 years of age and 0.55% of whom were over 95 years of age [11]. Several studies have highlighted the importance of the health problems and comorbidity associated with the aging population, such as cognitive impairment, cardiovascular diseases, degenerative pathologies, or cancer [12–17], and the need to address them from a comprehensive perspective of attention to disease, dependency and the provision of services and care for the LLIs. However, few authors have addressed the clinical and environmental factors associated with a healthy lifespan and the pathway for reducing the pathological consequences of ageing. The study ‘Genetic, environmental and lifestyle factors associated with longevity’ (LONGECYL Study) [18] aims to fully describe a representative population of nonagenarians-centenarians from the region of Castile and Leon from both the genetic and epidemiological points of view to identify the genetic background and environmental, lifestyle and socioeconomic factors related to their health status.

We designed a collaborative study among the Health Sentinel Network of Castile and Leon (HSNCyL) in Valladolid, the National DNA Bank (BNADN) at the University of Salamanca and the Human Genotyping Unit at the Spanish National Cancer Research Center (GU-CNIO) in Madrid with the following objectives: (1) to describe the health of the regional population aged *≥* 95; (2) to identify the environmental and lifestyle factors associated with their health status and quality of life; (3) to confirm/rule out already described genetic factors associated with longevity in the Spanish population; (4) to identify novel genetic factors related to longevity; (5) to study the interaction among genetic and nongenetic factors; and (6) to assess the epigenetic profile related to longevity.

This paper describes the clinical and perceived health status, quality of life, dependency for activities of daily living, and other social and demographic variables of the population that had reached the age of 95 years or more. We explore the factors associated with a better quality of life and low dependence to advise the health authorities and social services to guide the policy and programs addressed to LLI.

## Materials and methods

### Design

The HSNCyL is a health information system based on primary care doctors and nurses, with standardized methods in surveillance and epidemiological research [19], comprising a representative population living in the region [20].

The sentinel population covered by the HSNCyL in 2019 was 186,123 inhabitants, which included 1298 long-lived individuals (LLIs) whose 95th or higher birthday was between the 1st of March 2019 and the 28th of February 2020, representing 0.70% of the sentinel population (73% female; 76.6% < 98, 14.9% between 98 and 99, and 8.5% *≥*100 years old).

The design and main response achievements of this study have already been described [18]. Briefly, the sentinel doctors and nurses received a list of the 1298 LLI covered by the HSNCyL with their addresses and telephone numbers. The persons or their tutors were contacted to explain the study’s main objectives and to arrange a medical appointment to describe the research in detail and sign the written informed consent form.

Out of the 1298 LLI, 355 could not be contacted or were excluded because of death before the survey (66), declined to participate in the study (43), showed advanced cognitive impairment (5), were not located or not accessible (219) or other causes (22). The final sample was composed of 943 LLI, 72.7% of the eligible population. Only vital status and clinical information were obtained from the caregiver and clinical records for 184, and 759 LLIs were fully studied and interviewed.

### Variable definitions

The sentinel doctors and nurses filled in a standard interview questionnaire that included social and demographic information, medical background from the electronic medical record (eMR), clinical examination, anthropometric data, the EQ-5D-3 [21], lifestyle habits (including diet), working life, family health, and demographic history. A blood sample was drawn into an EDTA-collection tube or, alternatively, a saliva sample was collected to obtain DNA. For all patients, a hemogram was performed, and standard blood biochemistry was determined.

The chronic pathology included in the eMR was recorded, coded, and grouped into the major disease groups of the International Classification of Diseases, Tenth Revision (ICD-10). The number of diseases present at the time of the study in each LLI was also calculated.

Quality of life was measured according to the Spanish version of the EQ-5D-3 [22], and the global index of each health status was calculated using the algorithm and parameters for the Spanish population for each level and dimension, as described by Herdman et al. [23]. This quality-of-life index, ranging from 0 to 1, was categorized into less than 0.25, 0.25–0.49, 0.50–0.74 and 0.75-1.

Dependency was measured using the Barthel index, which classified it as independent or slight dependency, moderate dependency, severe dependency, and total dependency. Perceived health was classified, for comparison with national data, as excellent, very good, good, fair and poor.

Individuals were considered to be hypertensive if systolic blood pressure was equal to or greater than 140 or diastolic blood pressure was equal to or greater than 90 or if they had a history of hypertension or were being treated with antihypertensive drugs. They were considered to have diabetes mellitus if fasting blood glucose was equal to or greater than 126 mg/ml, if they had a history of diabetes mellitus, or if they were being treated with oral antidiabetics or insulin. They were considered hyperlipidemic if total cholesterol was equal to or greater than 200 mg/ml, if the patient had a history of hyperlipidemia, or if the patient was being treated with lipid-lowering drugs.

Body mass index (BMI) was stratified into underweight (< 21.9 kg/m^2^), normal weight (22–26.9 kg/m^2^), and overweight or obese (*≥* 27 kg/m^2^) according to the recommendations for the older population [24, 25]. Individuals were considered overweight when the BMI was *≥* 27 kg/m^2^ or if they were diagnosed to be overweight.

We considered individuals to be anemic when the hemoglobin value was equal to or less than 12 g/dL for women and 13 g/dL for men (as per the World Health Organization [26]). Similarly, renal function was categorized into three values (*≥* 60%, 30–59% and less than 30%) according to the Chronic Kidney Disease Epidemiology Collaboration (CKD-EPI) [27].

Standard Drink Units (SDUs) per week were calculated considering the glasses of wine and beer (1 SDU per glass), and spirits (2 SDUs per glass) consumed with meals and away from meals from Monday to Sunday. For the analysis, SDUs were stratified into three categories: 0, 1–15 and 16 and more SDUs per week.

We recorded who had been a smoker or nonsmoker/occasional daily smoker and the years of consumption. The pack-year index was calculated by multiplying the years of cigar and cigarette consumption by the number of units per day and dividing it by 20. For the analysis, the variable was stratified into 0 and 1 as no smoking history or daily or occasional smoking history, respectively.

### Statistical analysis

Relative frequency distributions were calculated for the different variables included in this study. For chronic clinical processes, all diseases diagnosed in the study population were counted, and the prevalence in this population was calculated per ICD-10 chapter.

Bivariate analysis of categorical variables versus quality of life (four ordinal categories) and dependency (four ordinal categories) was performed with Pearson’s chi-square test, excluding missing values. Variables with a statistical significance (*p* < 0.05) were selected for the logistic regression models.

In the two logistic regression models, the dependent variable ‘quality of life’ was classified as 1 (quality-of-life index of 0.5 and above) and 0 (quality-of-life index below 0.5), and the variable ‘dependency’ was classified as 1 (independency, slight or moderate dependency) and 0 (severe or total dependency).

The logistic regression models were developed in two phases. Firstly, a stepwise phase (with a minimum input significance of *p* < 0.05 and an output significance of *p* < 0.10) with age, sex, and number of diagnosed diseases presented by LLIs, regardless their significance in the bivariate analysis, and those statistically significant in the bivariate analysis, as independent variables. Age was stratified in three categories: 95–97; 98–99; 100 and more years old at the moment of the study. Number of diagnoses diseases was included as a continuous variable with two units of change for the ORs estimates. The rest of variables in the models were categorized with their respective reference values.

In the second phase, the models were adjusted with all significant variables of the first phase and the ORs were estimated with their 95% confidence intervals (CIs).

## Ethical considerations

In March 2018, the Clinical Research Ethics Committee of the Health Area of Valladolid Este (Spain) approved the protocol (PI024-18) and in November 2018, the HSNCyL Steering Committee included the study in the 2019 annual program. Doctors and nurses were instructed to inform the selected persons, their relatives, or caregivers about the objectives of the study, the procedures of the survey, the clinical examination, and the blood sampling. The person or legal guardian was asked to sign written consent for the survey, the examination, access to their medical record and blood sampling. They also consented to be contacted in the future for follow-ups or for receiving relevant clinical results.

Of the 1298 eligible long-lived individuals, 354 were excluded or could not be contacted for various reasons (terminal patient, death, relocation, or lack of consent). Of the 944 individuals studied, 759 completed the questionnaire, and these individuals made up the sample for this study.

## Results

Of the 759 LLIs included, 72.6% were women and 21.5% were 98 years of age or older (15.8% of men and 23.6% of women). Ninety-eight point 7% were born in Spain, and 90.8% were born in Castile and Leon; 58.1% had lived mainly in rural areas, and 60.6% of the men and 39.7% of the women had worked in the primary sector (agriculture and livestock).

### Perceived health, quality of life and dependency

Of the LLIs, 64.2% perceived their health as good, very good or excellent, with minimal differences between sexes; this number increased to 71.6% in the oldest LLIs, aged 100 years and over (Table 1). However, the percentage of LLIs with a quality-of-life Index equal to or greater than 0.5 was 46.0%. On the other hand, independence or moderate or mild dependency accounted for 44.1%, with higher levels of independence among men than among women.

**Table 1:** Perceived health, quality of life, health status and dependency by sex and age group

	Sex		Age at inclusion in the study			All (*n* = 759)	
	Male (*n* = 208)	Female (*n* = 551)	Less than 98 (*n* = 596)	98–99 (*n* = 110)	100 and over (*n* = 53)		
	%	%	%	%	%	n	%
**Perceived Health**							
** Excellent**	6.7	4.5	4.0	9.1	9.3	39	5.1
** Very good**	12.5	15.6	13.1	16.4	30.2	112	14.8
** Good**	46.6	43.4	46.5	38.2	32.1	336	44.3
** Fair**	27.4	27.8	28.0	30.9	17.0	210	27.7
** Poor**	4.8	7.4	7.4	3.6	5.7	51	6.7
** Unknown**	1.9	1.3	1.0	1.8	5.7	11	1.4
**Quality-of-life index (from 0 to 1)**							
** 0.75 and over**	22.1	8.0	12.4	10.9	7.5	90	11.9
** From 0.50 to 0.74**	35.1	33.8	33.1	33.6	47.2	259	34.1
** From 0.25 to 0.49**	15.4	14.0	15.6	12.7	3.8	109	14.4
** Less than 0.25**	27.4	44.3	38.9	42.7	41.5	301	39.7
**Difficulty with near vision**	43.8	46.5	46.5	45.5	37.7	347	45.7
**Difficulty with distance vision**	36.1	33.9	35.7	29.1	32.1	262	34.5
**Difficulty hearing**							
** None**	15.4	18.7	18.3	17.3	13.2	135	17.8
** Some**	31.3	29.4	30.0	31.8	24.5	227	29.9
** Moderate**	33.2	29.6	31.7	22.7	34.0	232	30.6
** Severe**	18.3	20.9	18.8	24.5	26.4	153	20.2
** Unknown**	1.9	1.5	1.2	3.6	1.9	12	1.6
**Use of hearing aid**	18.3	16.0	16.9	19.1	7.5	126	16.6
**Sleep quality**							
** Very good**	17.3	17.2	16.9	18.2	18.9	131	17.3
** Good**	42.8	39.7	40.3	38.2	49.1	308	40.6
** Fair**	26.9	28.5	28.7	27.3	22.6	213	28.1
** Poor**	8.2	9.1	8.7	10.0	7.5	67	8.8
** Very poor**	2.4	2.7	3.0	1.8	.	20	2.6
** Unknown**	2.4	2.7	2.3	4.5	1.9	20	2.6
**Oral Health**							
** Very good**	5.8	4.5	4.4	6.4	7.5	37	4.9
** Good**	38.5	39.9	39.4	35.5	49.1	300	39.5
** Fair**	23.1	30.5	28.5	30.0	24.5	216	28.5
** Poor**	22.1	16.7	19.3	16.4	9.4	138	18.2
** Very poor**	4.8	4.0	4.2	4.5	3.8	32	4.2
** Unknown**	5.8	4.4	4.2	7.3	5.7	36	4.7
**Difficulty eating**							
** Much**	2.4	3.8	3.9	1.8	1.9	26	3.4
** Considerably**	12.0	11.6	12.6	10.9	3.8	89	11.7
** Some**	34.1	35.6	33.4	36.4	52.8	267	35.2
** None**	47.1	45.0	46.6	45.5	34.0	346	45.6
** Unknown**	4.3	4.0	3.5	5.5	7.5	31	4.1
**Barthel Index**							
** Independent/ Slight dependency**	14.9	7.3	9.4	10.9	5.7	71	9.4
** Moderate dependency**	39.9	32.7	36.4	25.5	34.0	263	34.7
** Severe dependency**	29.3	34.1	32.4	41.8	18.9	249	32.8
** Total dependency**	15.9	26.0	21.8	21.8	41.5	176	23.2
**Cognitive impairment**							
** No**	48.6	38.7	43.5	35.5	30.2	314	41.4
** Slight**	26.0	24.0	24.2	29.1	18.9	186	24.5
** Moderate or severe**	20.2	31.9	27.0	30.0	45.3	218	28.7
** Unknown**	5.3	5.4	5.4	5.5	5.7	41	5.4

Among the factors that can influence health, quality of life and dependence, it should be noted that more individuals had difficulty with near vision than with distance vision (45.7% and 34.5%, respectively). Hearing was not a major problem in more than half of the LLIs, and 16.6% used hearing aids.

Sleep quality was good or very good in a high percentage of the population (57.9%), with no differences according to age or sex. Oral health was sufficiently preserved so that 45.6% had no difficulty eating.

Finally, moderate or severe cognitive impairment was observed to be greater among women (31.9%) than among men (20.2%) and increased with age to 45.3% in those over 100 years of age.

### Prevalence of diseases

A total of 3,493 chronic diseases were recorded in the population, corresponding to 4.6 diseases per LLI (4.8 in men and 4.5 in women, *p* < 0.05); there was a decreasing trend with age, from 4.7 in those under 98 vs. 3.3 in those over 100 (*p* < 0.05).

The prevalence of LLIs with diseases of the circulatory system was estimated at 85.5%; it was higher among women (86.9%) and decreased slightly with age (Table 2). This was followed by endocrine, nutritional, and metabolic diseases, with a prevalence of 45.7%, which was also higher among women (47.7%) than among men (40.4%). Next on the list were diseases of the musculoskeletal system and connective tissue and diseases of the genitourinary system, with marked differences according to gender: there was a higher prevalence among women of the former and among men of the latter diseases. The prevalence by age did not, in general, show a linear increase. Rather, in some disease groups, the prevalence decreased in the group of individuals over 100 years of age.

**Table 2:** Prevalence of diseases present in the LLI population

	Sex		Age at inclusion in the study			All (*n* = 759)
	Male (*n* = 208)	Female (*n* = 551)	Less than 98 (*n* = 596)	98–99 (*n* = 110)	100 and over (*n* = 53)	
	%	%	%	%	%	%
**Diseases of the circulatory system**	81.7	86.9	85.9	84.6	83.0	85.5
**Endocrine, nutritional and metabolic diseases**	40.4	47.7	48.8	34.6	34.0	45.7
**Diseases of the musculoskeletal system and connective tissue**	29.8	43.6	39.9	40.9	35.9	39.8
**Diseases of the genitourinary system**	45.2	20.9	28.4	29.1	15.1	27.5
**Diseases of the digestive system**	23.6	19.2	20.6	25.5	7.6	20.4
**Mental and behavioral disorders**	11.1	18.7	17.3	12.7	17.0	16.6
**Diseases of the nervous system**	13.5	16.7	15.6	18.2	13.2	15.8
**Neoplasms**	22.6	11.1	14.4	10.9	18.9	14.2
**Diseases of the respiratory system**	16.4	11.1	12.3	13.6	13.2	12.5
**Other diseases**	55.3	51.4	52.9	53.6	45.3	52.4

Regarding the principal cardiovascular risk factors, the prevalence of arterial hypertension was very high (85.2%), while diabetes was estimated at 21.5% and hypercholesterolemia at 37.3% (Table 3). Weight was preserved, with overweight or obesity in 44.5%, whereas 14.4% of the men and 18.7% of the women were underweight. Thirty-three point 6% had some degree of anemia, and a severe decrease in glomerular filtration rate affected only 10% of the LLIs.

**Table 3:** Clinical assessment of the ILLs: Cardiovascular risk history, examination and laboratory tests

	Sex		Age at inclusion in the study			All (*n* = 759)	
	Male (*n* = 208)	Female (*n* = 551)	Less than 98 (*n* = 596)	98–99 (*n* = 110)	100 and over (*n* = 53)		
	%	%	%	%	%	n	%
**Arterial hypertension (altered blood pressure, history or treatment)**	79.8	87.3	86.1	81.8	83.0	647	85.2
**Diabetes mellitus (impaired fasting glucose, history or treatment)**	19.7	22.1	22.5	17.3	18.9	163	21.5
**Hypercholesterolemia (abnormal cholesterol, history or treatment)**	26.0	41.6	39.4	34.5	18.9	283	37.3
**Current body mass index**							
** Malnourished/underweight**	14.4	18.7	18.3	13.6	16.7	133	17.5
** Normal weight**	40.4	29.3	31.9	35.5	31.5	246	32.4
** Overweight/obese**	27.4	32.2	32.4	28.2	20.4	235	30.9
** Unknown**	17.8	19.7	17.4	22.7	31.5	146	19.2
**Overweight and obesity (altered BMI or history)**	43.3	45.0	45.3	45.5	34.0	338	44.5
**Change from adult weight**							
** Same**	39.4	37.3	37.1	39.1	44.4	288	37.9
** Increased**	35.1	28.4	30.9	32.7	18.5	230	30.3
** Decreased**	21.6	30.4	29.2	20.0	31.5	213	28.0
** Unknown**	3.8	3.8	2.9	8.2	5.6	29	3.8
**Smoking history**	51.4	2.0	16.8	11.8	9.4	118	15.5
**Consumption of Standard Drink Units per week**							
** None**	30.0	66.6	57.9	58.2	69.8	446	58.8
** From 1 to 15**	18.3	20.5	20.3	20.0	15.1	151	19.9
** 16 or more**	39.9	8.0	17.4	15.5	11.3	127	16.7
** Unknown**	3.8	4.9	4.4	6.4	3.8	35	4.6
**Anemia**	39.4	31.3	33.1	33.6	38.9	255	33.6
**Renal function CKD-EPI**							
** ≥** **60%**	30.3	30.1	32.9	21.8	16.7	229	30.1
** 30–59%**	42.3	35.5	37.6	38.2	33.3	284	37.4
** < 30**	8.2	10.7	9.4	10.9	14.8	76	10.0
** Unknown**	19.2	23.7	20.1	29.1	35.2	171	22.5

### Factors associated with quality of life and dependency

Men had a better quality of life than women (*p* < 0.05) and greater independence in activities of daily living: 14.9% of men had independence or slight dependence compared to 7.3% of women (*p* < 0.05) (Table 4). For age, no variation in quality of life was observed, but an increase in dependency was observed (*p* < 0.05). Of the other variables studied, it is worth noting that the quality of life (*p* < 0.05) and independence (*p* < 0.05) were better in those who live alone, in those who do not have a permanent caregiver or whose caregiver is their partner (*p* < 0.05) and, of course, that there was no cognitive deterioration (*p* < 0.05).

**Table 4:** Factors associated with quality of life and dependency of the LLIs

	Overall quality-of-life index (from 0 to 1)					Barthel Index					Total
	0.75 and over	0.50 to 0.74	0.25 to 0.49	Less than 0.25		Indep. or slight dependency	Moderate dependency	Severe dependency	Total dependency		
	%	%	%	%	p*	%	%	%	%	p*	*n*
**All**	11.9	34.1	14.4	39.7		9.4	34.7	32.8	23.2		759
**Sex**					< 0.0001					0.0002	
** Male**	22.1	35.1	15.4	27.4		14.9	39.9	29.3	15.9		208
** Female**	8.0	33.8	14.0	44.3		7.3	32.7	34.1	26.0		551
**Age at inclusion in the study**					0.1578					0.0053	
** Less than 98**	12.4	33.1	15.6	38.9		9.4	36.4	32.4	21.8		596
** 98–99**	10.9	33.6	12.7	42.7		10.9	25.5	41.8	21.8		110
** 100 and over**	7.5	47.2	3.8	41.5		5.7	34.0	18.9	41.5		53
**Lifetime residence category**					0.0322					0.1312	
** Rural**	10.2	31.5	15.6	42.6		7.9	34.5	32.4	25.2		441
** Urban**	14.4	38.0	12.8	34.8		11.8	35.7	33.1	19.3		305
** Unknown**	7.7	30.8	7.7	53.8		.	15.4	38.5	46.2		13
**Current residence**					< 0.0001					< 0.0001	
** At home with their family**	13.9	30.7	16.2	39.2		9.7	34.3	36.6	19.4		309
** At home alone**	20.7	45.0	14.4	19.8		24.3	47.7	22.5	5.4		111
** At their family’s home**	7.4	40.2	13.9	38.5		4.9	42.6	29.5	23.0		122
** In a nursing home**	6.7	29.5	11.4	52.4		3.8	23.8	33.3	39.0		210
** Unknown**	14.3	42.9	28.6	14.3		.	28.6	71.4	.	0.2105	7
**Marital status**					0.1149						
** Single**	9.0	34.3	19.4	37.3		9.0	32.8	34.3	23.9		67
** Married / partner**	21.4	30.1	15.5	33.0		16.5	35.0	34.0	14.6		103
** Widowed**	10.7	35.1	13.6	40.6		8.3	35.2	32.1	24.4		579
** Separated / divorced**	.	100.0	.	.		.	100.0	.	.		1
** Unknown**	.	11.1	11.1	77.8		.	.	55.6	44.4		9
**Type of caregiver (simplified)**					< 0.0001					< 0.0001	
** No permanent caregiver**	38.5	53.8	5.1	2.6		46.2	48.7	2.6	2.6		39
** Partner**	35.3	23.5	23.5	17.6		23.5	29.4	41.2	5.9		17
** Children**	13.6	36.3	14.2	36.0		10.8	38.5	32.3	18.4		353
** Paid caregiver**	4.1	30.6	20.7	44.6		2.5	36.4	40.5	20.7		121
** In a nursing facility**	6.7	29.5	11.4	52.4		3.8	23.8	33.3	39.0		210
** Unknown**	10.5	36.8	21.1	31.6		.	47.4	42.1	10.5		19
**Cognitive impairment**					< 0.0001					< 0.0001	
** No**	22.3	44.6	12.4	20.7		16.9	54.5	23.6	5.1		314
** Mild**	8.6	41.4	16.1	33.9		7.0	32.3	45.2	15.6		186
** Moderate or severe**	0.9	12.8	15.6	70.6		0.9	11.5	33.9	53.7		218
** Unknown**	4.9	34.1	14.6	46.3		7.3	17.1	41.5	34.1		41
**Difficulty with near vision**					< 0.0001					0.0001	
** Yes**	6.1	28.2	17.9	47.8		6.6	31.7	34.9	26.8		347
** No**	18.3	42.0	12.1	27.5		12.7	40.2	31.0	16.2		371
** Unknown**	2.4	12.2	4.9	80.5		2.4	9.8	31.7	56.1		41
**Difficulty with distance vision**					< 0.0001					< 0.0001	
** Yes**	8.0	24.0	14.9	53.1		6.1	25.2	36.3	32.4		262
** No**	15.4	42.6	15.2	26.7		12.2	43.5	31.1	13.1		434
** Unknown**	3.2	17.5	6.3	73.0		3.2	12.7	30.2	54.0		63
**Difficulty hearing**					0.0083					0.0308	
** None-some**	15.5	36.5	13.3	34.8		11.9	37.0	31.5	19.6		362
** Moderate-severe**	8.8	32.5	15.3	43.4		7.0	33.2	34.0	25.7		385
** Unknown**	.	16.7	16.7	66.7		8.3	8.3	33.3	50.0		12
**Sleep quality**					0.0275					0.1661	
** Very good or good**	14.8	35.1	14.4	35.8		11.6	34.4	32.6	21.4		439
** Fair-very poor**	8.0	34.3	15.0	42.7		6.7	37.0	33.7	22.7		300
** Unknown**	5.0	10.0	5.0	80.0		.	5.0	25.0	70.0		20
**Type of smoker**					0.0074					0.0015	
** Never smoked**	10.8	32.8	14.5	42.0		8.7	32.9	32.8	25.6		641
** Occasional or daily smoker**	17.8	41.5	13.6	27.1		12.7	44.1	33.1	10.2		118
**Consumption of Standard Drink Units per week**					0.0013					0.0432	
** None**	9.6	30.7	15.2	44.4		8.5	32.3	34.1	25.1		446
** 1 to 15**	15.9	39.7	12.6	31.8		10.6	41.1	27.2	21.2		1
** 16 or more**	17.3	40.2	15.7	26.8		12.6	38.6	35.4	13.4		127
** Unknown**	2.9	31.4	5.7	60.0		2.9	22.9	31.4	42.9		35
**Hypertension or treatment or history**					0.6816					0.9717	
** Yes**	11.9	35.1	14.2	38.8		9.6	35.4	33.2	21.8		647
** No**	16.9	32.4	14.1	36.6		8.5	35.2	32.4	23.9		71
** Unknown**	2.4	22.0	17.1	58.5		7.3	22.0	26.8	43.9		41
**Altered blood glucose or history**					0.0111					0.0135	
** Yes**	7.4	28.2	18.4	46.0		6.1	29.4	33.7	30.7		163
** No**	13.5	36.4	13.7	36.4		10.4	37.1	32.3	20.1		517
** Unknown**	10.1	31.6	10.1	48.1		8.9	29.1	34.2	27.8		79
**Altered cholesterol or history**					0.6753					0.0549	
** Yes**	13.8	36.0	15.2	35.0		10.6	39.2	33.2	17.0		283
** No**	11.8	34.1	14.7	39.4		8.7	33.3	32.5	25.5		381
** Unknown**	6.3	28.4	10.5	54.7		8.4	26.3	32.6	32.6		95
**Altered BMI or history of overweight**					0.1185					0.0229	
** Yes**	14.5	40.5	11.8	33.1		10.7	41.7	32.8	14.8		338
** No**	12.8	32.7	15.7	38.8		10.0	34.2	31.7	24.2		281
** Unknown**	3.6	21.4	17.9	57.1		5.0	18.6	35.0	41.4		140
**Anemia**					0.0003					< 0.0001	
** Yes**	7.1	30.2	16.5	46.3		29.8	35.3	29.8	29.8		255
** No**	16.0	36.0	14.8	33.3		38.9	31.5	17.5	24.2		406
** Unknown**	7.1	36.7	7.1	49.0		29.6	31.6	29.6	41.4		98
**Renal function CKD-EPI**					0.3745					0.4451	
** ≥** **60%**	10.0	34.1	14.4	41.5		8.3	31.4	36.7	23.6		229
** 30–60%**	16.5	33.1	14.1	36.3		12.3	37.3	28.9	21.5		284
** < 30**	10.5	32.9	21.1	35.5		6.6	32.9	40.8	19.7		76
** Unknown**	7.1	36.5	11.8	44.7		7.1	35.3	30.6	27.1		170
**Diseases of the circulatory system**					0.0044					0.2566	
** Yes**	10.3	35.6	14.9	39.1		8.6	34.8	33.7	22.8		649
** No**	20.9	25.5	10.9	42.7		13.6	33.6	27.3	25.5		110
**Diseases of the respiratory system**					0.2670					0.2369	
** Yes**	6.3	40.0	13.7	40.0		5.3	30.5	40.0	24.2		95
** No**	12.7	33.3	14.5	39.6		9.9	35.2	31.8	23.0		664
**Diseases of the genitourinary system**					0.0792					0.0654	
** Yes**	15.3	35.9	15.8	33.0		11.5	40.2	27.3	21.1		209
** No**	10.5	33.5	13.8	42.2		8.5	32.5	34.9	24.0		550
**Endocrine, nutritional and metabolic diseases**					0.7061					0.9240	
** Yes**	10.7	35.7	14.7	38.9		9.5	35.7	31.7	23.1		347
** No**	12.9	32.8	14.1	40.3		9.2	33.7	33.7	23.3		412
**Neoplasms**					0.0630					< 0.0001	
** Yes**	17.6	36.1	16.7	29.6		20.4	33.3	34.3	12.0		108
** No**	10.9	33.8	14.0	41.3		7.5	34.9	32.6	25.0		651
**Diseases of the musculoskeletal system**					0.0269					0.2012	
** Yes**	7.6	36.4	13.9	42.1		8.6	36.8	35.1	19.5		302
** No**	14.7	32.6	14.7	38.1		9.8	33.3	31.3	25.6		457
**Diseases of the digestive system**					0.6254					0.5886	
** Yes**	10.3	31.0	15.5	43.2		7.1	32.9	34.2	25.8		155
** No**	12.3	34.9	14.1	38.7		9.9	35.1	32.5	22.5		604
**Diseases of the nervous system**					< 0.0001					< 0.0001	
** Yes**	5.0	24.2	12.5	58.3		3.3	24.2	30.8	41.7		120
** No**	13.1	36.0	14.7	36.2		10.5	36.6	33.2	19.7		639
**Mental and behavioral disorders**					< 0.0001					< 0.0001	
** Yes**	3.2	19.8	15.1	61.9		4.0	25.4	27.8	42.9		126
** No**	13.6	37.0	14.2	35.2		10.4	36.5	33.8	19.3		633

*Chi Square significance excluding missing values in each category

Both near and far vision and hearing were also significantly associated with quality of life and independence, as was the quality of sleep. Although smoking history was associated with worse quality of life and dependency, the same was not true for a history of alcohol consumption, which showed a significant inverse association.

Finally, the diseases that were associated with worse quality of life were diseases of the circulatory system, musculoskeletal system, nervous system, and mental and behavioral disorders; diseases associated with greater dependency were neoplasms, diseases of the nervous system, and mental and behavioral disorders. The presence of anemia or diabetes was also associated with a lower quality of life and greater dependency, whereas obesity only had an effect on dependency.

Logistic regression models (Table 5) showed that quality of life was better in individuals over 100 years of age (OR 7.98; CI: 2.32–27.41) than in those aged 95 to 97 and that independence was greater in men (OR 2.43; CI: 1.40–4.29) than in women. Living at home and alone, having no paid caregiver or living with one’s children was associated with better quality of life and greater independence. Consumption of one or more SDUs per week presented protective ORs, although they did not reach significance.

**Table 5:** Logistic regression models. A: Quality-of-life index *≥* 0.5; B: Independence, mild or moderate dependency/severe or total dependency

Effect	Model Quality-of-life index *≥* 0.5	Model independence, mild or moderate dependency/severe or total dependency
	OR (CI)	OR (CI)
Age at inclusion		Not in the model
Less than 98	1	
98–99	1.35 (0.70–2.64)	
100 and over	7.98 (2.32–27.41)	
Sex	Not in the model	
Female		1
Male		2.43 (1.40–4.29)
Current residence		
At their family’s home	1	1
At home with their family	1.25 (0.66–2.37)	0.59 (0.31–1.13)
At home alone	3.00 (1.18–7.62)	2.43 (0.93–6.39)
In a nursing home	2.71 (0.99–7.39)	0.84 (0.31–2.28)
Type of caregiver		
Paid caregiver	1	1
No permanent caregiver	28.29 (3.42-233.92	14.92 (1.76-127.01)
Partner	3.21 (0.70-14.66	1.00 (0.28–4.60)
Children	2.57 (1.27–5.20	1.93 (0.95–3.94)
Cognitive impairment		
Moderate-severe	1	1
No cognitive deficit	7.32 (3.51–15.28)	10.65 (5.39–21.04)
Mild cognitive impairment	3.12 (1.47–6.61)	2.31 (1.15–4.66)
Difficulty with near vision		Not in the model
Yes	1	
No	2.12 (1.21–3.73)	
Difficulty with distance vision		Not in the model
Yes	1	
No	1.98 (1.10–3.59)	
Anemia		
Yes	Not in the model	1
No		1.78 (1.09–2.99)
SDU consumption per week		
16 or more	1	Not in the model
None	0.36 (0.19–0.69)	
1 to 15	0.87 (0.40–1.89)	
Nervous_system_diseases		
Yes	1	Not in the model
No	1.49 (0.72–3.08)	
Mental_and_behavioral_disorders		Not in the model
Yes	1	
No	2.44 (1.20–4.92	

OR: Odds Ratio estimates; CI: Confidence interval 95%; SDU: Standard Drink Units

Regarding health problems, not having difficulties with near vision (OR 2.12; CI: 1.21–3.73) or distance vision (OR 1.98; CI: 1.10–3.59) was associated with better quality of life. Individuals with anemia had worse values for dependency. Finally, individuals with no or mild cognitive impairment had ORs of 7.32 (CI: 3.51–15.28) and 3.12 (CI: 1.47–6.61), respectively, of having an above-average quality of life compared to those with moderate or severe cognitive impairment. Mental and behavioral disorders also had a significant influence on quality of life.

## Discussion

The present study allows us to make an overall assessment of the situation facing the social and healthcare system and society in general due to population aging and the increase in the number of LLIs. More than 13,000 LLIs live in Castile and Leon, with a more rural than urban distribution, which has an effect on their social and health situation and the provision of adequate services.

Sixty-four point 2% of LLIs perceived their health as good, very good or excellent, a percentage that exceeded that recorded in the 2017 National Health Survey (ENS2017) [28] for all Spaniards over 85 years of age, which was 32.4%, and also higher than the 55.7% of good and very good perceived health reported in Barcelona in LLI aged 95 and more [29]. As in the ENS2017, men had a slightly better perception of their health than women, and surprisingly, this perception was higher in the oldest. This has also been observed in other studies, where patients with multiple pathologies younger than 65 have a worse health-related quality of life (measured with the EQ-5D) than those over 65 [30], which could be related to lower comorbidity, lower drug intake, and greater autonomy in older people [29].

The quality of life index (ranging from 0 to 1) improved with age and showed more differences by sex, with a better quality of life in men, which is consistent with one European study in which women had lower quality of life than men, particularly in Southern countries [31]. Overall, 46% had an index equal to or higher than 0.5. Finally, independence and slight or moderate dependence accounted for 45.1%. In general, it can be said that the quality of life was consistent with the degree of dependency but that the perception of health status was better.

The prevalence of chronic pathology was significantly higher in men than in women and decreased with age, which would support an improvement in the quality of life in older population groups. Diseases of the circulatory system were present in 4 out of 5 LLIs; they were mainly accounted for by arterial hypertension, with a prevalence of 85.2%, which was more than double that observed in the population of Castile and Leon over 15 years of age in 2004 (38.7%) but only slightly more than that of those over 75 years of age (76.3%) [32]. Men presented more genitourinary and digestive pathologies, and women suffered more from mental disorders and diseases of the nervous system, which reduced their quality of life compared to men. Diabetes was present in 21.5% of the LLIs, compared to 9.9% in those over 15 years of age and 21.0% in those over 75 years of age in Castile and Leon in 2004 [33].

Age was positively correlated with a better quality of life, as did not having cognitive deficits, having good near and far vision, and not suffering from nervous system or mental illnesses. Alcohol consumption correlated, albeit not significantly, with a better quality of life, which may be related to subjective perceptions. Smoking did not show any correlation in the multilogistic models, perhaps because of the limitation of recall or because the years in the ex-smoker category were not measured with sufficient accuracy. Logically, living alone at home and not having a permanent caregiver were also associated with better quality of life.

Regarding dependency, age neither increased nor decreased it, but being male and not suffering from cognitive impairment was associated with greater independence. Similar to quality of life, living alone and without a caregiver was also associated with greater independence. The only significant biological parameter in the multilogistic model was anemia, which was associated with greater dependency.

A recent study in Valencia (Spain) concluded that successful aging in people over 95 years of age is characterized by good perceived and cognitive health, a good family social support network, a history of long-lived family members, healthy lifestyles and participation in various activities [34]. The data from Castile and Leon show that LLIs have a relatively well-preserved health status compared to the preceding age groups and perceive it as such, possibly because of the selective effect of mortality of people with severe multiple pathologies, as has been previously described [35, 36]. Mental illnesses and diseases of the nervous system, which have a higher survival and therefore prevalence above 95 years of age, are the ones that will most condition the quality of life and dependence of this population.

At the opposite extreme would be frailty as a greater vulnerability and a lower capacity to respond to the situation of presenting with multiple pathologies [12]. In the present study, although frailty was not specifically analyzed, mortality in the first year of follow-up (until March 1, 2021) was 54.5% in men and 45.7% in women [37], although the possible effect of the COVID-19 pandemic on excess mortality should be borne in mind.

One of the limitations of this study was the difficulty of accessing the reference population because of age, moving residence and the assumed limited life expectancy. The number of subjects who declined to participate within the study represents only 5%, and most of the LLI not studied were due to the change of residence (to a care home or children’s home) or death before their 95th birthday. There were nonsignificant differences by sex between the 759 LLI fully studied and the rest of the sample, which could not be interviewed to assess the quality of life (184), but the mean age was slightly lower, 96.4 and 96.7, respectively.

The present article leaves open some questions about the variables that favor better adaptation of the older persons to advanced disease. Neither the diet nor the working conditions of the people studied were included in this study, nor was the effect that some hardships suffered by the different cohorts, including for example, the Spanish Civil War and the postwar period, may have had. The models also did not include the socioeconomic level, the longevity history of ancestors nor, of course, the genetic variants of this population, which will be the subject of future analyses of this project.

These data provide relevant information about the health situation of LLI in the community and the main factors which contribute to a better health status. Public health interventions can be established to reduce the risk associated to a poor quality of life and disability, and social and health measures could be implemented to maintain the LLIs in good shape and with the greatest autonomy.

This study confirms that primary care is an extraordinary place for research on ageing. Sentinel doctors and nurses have the methodological skills and direct access to this population to achieve the highest response rate and to get the collaboration of the older persons and their relatives. This approach opens the door to new investigations in the field of clinical or public health intervention assessment in the LLI.

## Data Availability

The datasets used and analysed during the current study are available from the corresponding author on reasonable request.
